# Soritesidine, a Novel Proteinous Toxin from the Okinawan Marine Sponge *Spongosorites* sp.

**DOI:** 10.3390/md17040216

**Published:** 2019-04-08

**Authors:** Ryuichi Sakai, Kota Tanano, Takumi Ono, Masaya Kitano, Yusuke Iida, Koji Nakano, Mitsuru Jimbo

**Affiliations:** 1 Faculty and Graduate School of Fisheries Sciences, Hokkaido University, Sapporo, Hokkaido 060-0808, Japan; masa.northern@gmail.com (M.K.); rath_t-plos@eis.hokudai.ac.jp (Y.I.); sc.gg21st@gmail.com (K.N.); 2 School of Marine Bioscience, Kitasato University, Minato City, Tokyo 108-0072, Japan; tanano0927@gmail.com (K.T.); jiroukaja2323@gmail.com (T.O.); mjinbo@kitasato-u.ac.jp (M.J.)

**Keywords:** sponge, protein, cytotoxicity, mouse lethality, brine shrimp, genotoxin

## Abstract

A novel protein, soritesidine (SOR) with potent toxicity was isolated from the marine sponge *Spongosorites* sp. SOR exhibited wide range of toxicities over various organisms and cells including brine shrimp (*Artemia salina*) larvae, sea hare (*Aplysia kurodai*) eggs, mice, and cultured mammalian cells. Toxicities of SOR were extraordinary potent. It killed mice at 5 ng/mouse after intracerebroventricular (i.c.v.) injection, and brine shrimp and at 0.34 µg/mL. Cytotoxicity for cultured mammalian cancer cell lines against HeLa and L1210 cells were determined to be 0.062 and 12.11 ng/mL, respectively. The SOR-containing fraction cleaved plasmid DNA in a metal ion dependent manner showing genotoxicity of SOR. Purified SOR exhibited molecular weight of 108.7 kDa in MALDI-TOF MS data and isoelectric point of approximately 4.5. *N*-terminal amino acid sequence up to the 25th residue was determined by Edman degradation. Internal amino acid sequences for fifteen peptides isolated from the enzyme digest of SOR were also determined. None of those amino acid sequences showed similarity to existing proteins, suggesting that SOR is a new proteinous toxin.

## 1. Introduction

Marine sponges are known to be rich sources of novel bioactive peptides [1,2]. They are mostly small cyclic molecules (molecular weight, MW < 2000) composed of non-proteinous amino acids or even non-amino acid components, and thus are thought to be of non-ribosomal origin. There are, however, several examples of middle sized (MW < 6000) ribosomal peptides including polytheonamides [3], asteropines and related molecules [4,5], and aculeines [6] are known. These molecules are highly post-translationally modified by extensive methylation, disulfide bonds formation and polyamine addition, respectively. The unique modifications may add extra stability and structural characteristics to the molecules which in turn lead to specific and potent biological activities of the molecules. Besides these relatively small peptides, sponges are known to contain large ‘bioactive’ proteins of which apparent biological activity parallels with those of secondary metabolites. Such examples include cytotoxic, antiviral, antimicrobial, hemolytic or hemagglutinating proteins (lectins) reported in various sponges [7,8,9,10,11,12]. Occurrences of those molecules suggest that sponges have evolved to produce proteins not only with physiological but also with ecological functions. Since proteins or peptides with unique biological function can be engineered to produce biomedically useful compounds [13,14], sponges can be viewed as interesting source of novel functional polypeptides. In a course of our study to discover novel water soluble bioactive molecules [15,16,17] we found that an aqueous extract of *Spongosorites* sp., possessing cytotoxicity against cancer cell lines and potent lethality to brine shrimp larvae, contained the 110 kDa protein as the active principal. We describe here isolation, biological activities, and physico-chemical characterization of a novel marine toxin, soritesidine (SOR). 

## 2. Results

### 2.1. Extraction and Separation of the Sponge Metabolites

An aqueous extract of sponge specimen collected in Iriomote, Okinawa and identified to be *Spongosorites* sp. showed conspicuously potent cytotoxicity against three cancer cell lines and brine shrimp larvae. The cytotoxicity was somewhat cell line selective as HT-29 colon carcinoma cells were less sensitive than A549 lung carcinoma/MDA-MB-231 breast cancer cell lines (Table 1). 

The toxicity of the extract against brine shrimp was characterized by its slow onset, in that the activity was observed later than 24 h after inoculation of the extract to the media. To characterize the active principal, the aqueous extract was first separated on a Sephadex LH-20 column into a total of 38 fractions. The activity was observed near the void volume fractions 2–9, suggesting that the active molecule was larger than the exclusion limit of the gel. We also found that insoluble precipitation formed gradually in the active fractions upon standing at room temperature. These observations together suggested that the active component is a large biomolecule. 

**Figure 1:**
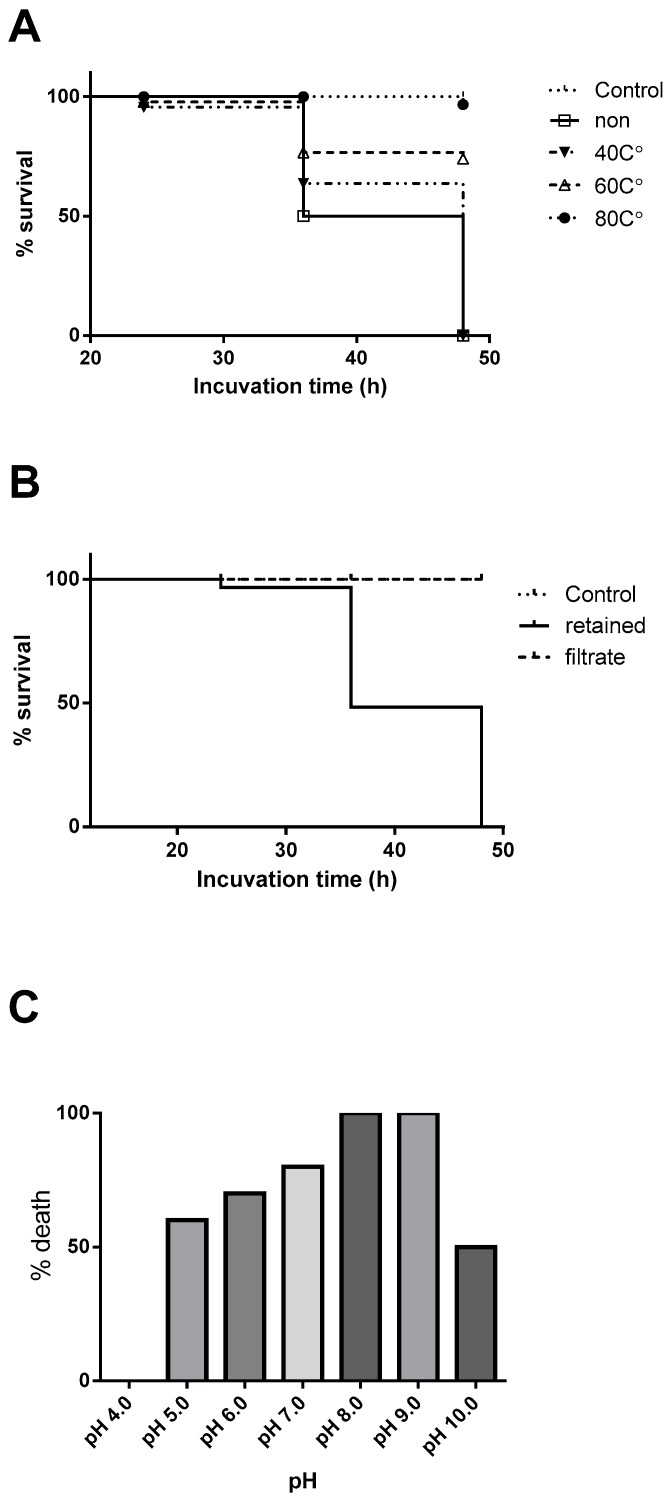
(**A**) Brine shrimp lethality of the crude extract after heat treatments, (**B**) ultrafiltration fraction using 30 kDa membrane, (**C**) treated in different buffers with various pH. All animals survived in both control and the filtrate groups.

We thus tested the stability of the active constituents by heating the extract at 40, 60, and 80 °C for 10 min and then tested for the brine shrimp lethality. The sample heated at 80 °C lost the activity completely, while the lower temperature groups lost the activity only partially (Figure 1A). We next assessed the size of the active molecules by ultrafiltration. The activity resided in a retained fraction upon an ultrafiltration of the extract using 30 kDa-cut membrane (Figure 1B). When the active fraction was treated in buffers with different pH, activity was retained in high pH buffers but was lost completely at pH ≤ 4 (Figure 1C). All above observation assured the proteinous nature of the active principal.

**Figure 2:**
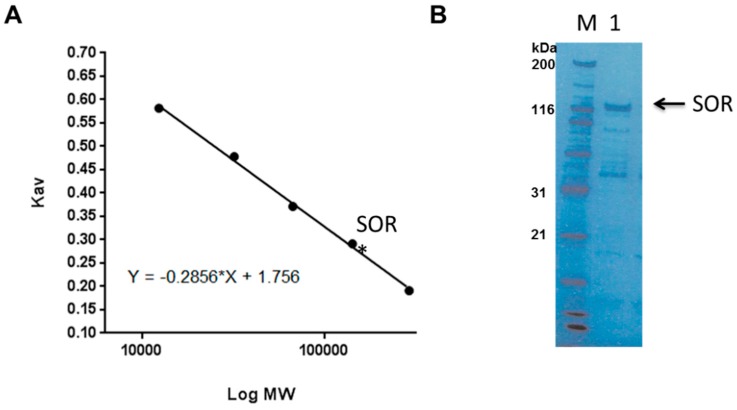
(**A**) An elution of the active soritesidine (SOR) in a size exclusion chromatography (Superdex 200–300). (**B**) SDS-PAGE for Fraction A; M, size marker, 1, Fraction A.

During the above experiment, we found that the sponge specimen, originally purple in color, turned black immediately after the collection and the extract irreversibly stained the Sephadex gel during the separation. Since this observation suggested a presence of tyrosinase and its substrate in the extract leading to formation of melanin, we extracted the sponge specimen using a buffer containing kojic acid, a tyrosinase inhibitor, and found that the additive attenuated the tanning of the extract without affecting its bioactivity. The sponge was extracted with a kojic acid-containing buffer. Next, chromatographic behavior of the active constituent was explored by using a combination of hydrophobic chromatography, anion exchange chromatography and gel filtration (Figure S1). When the crude extract was separated by a hydrophobic pre-packed HiTrap Butyl FF column with gradient of 1 M sodium sulfate and saline, the active component was eluted conductivity range between 140 and 40 mS/cm. The active fraction was further separated on RESOURCE-ISO with a gradient of 2 M sodium sulfate and saline to give the active fractions with the conductivity between 160 and 130 mS. In an anion exchange chromatography using RESOURCE-Q, the activity was eluted along the conductivity between 22.5 and 25.0 mS/cm. Finally this material was separated on a gel filtration chromatography to give active material at elution volume of 12.3 mL (chromatograms are shown in Figure S1). SDS-PAGE of the final product Fraction A, from the above separation, contained major bands at 120 kDa and few other impurities. Molecular size of the active protein was estimated to be 145 kDa in the gel filtration chromatography (Figure 2). Toxicity of the above fractions as indicated by the brine shrimp lethality increased about 600-fold from the crude extract (Table 2). Since a band at 120 kDa was concentrated drastically as the separation proceeds, it was thought to be the prime candidate for the toxic principal. 

### 2.2. Purification of SOR

**Figure 3:**
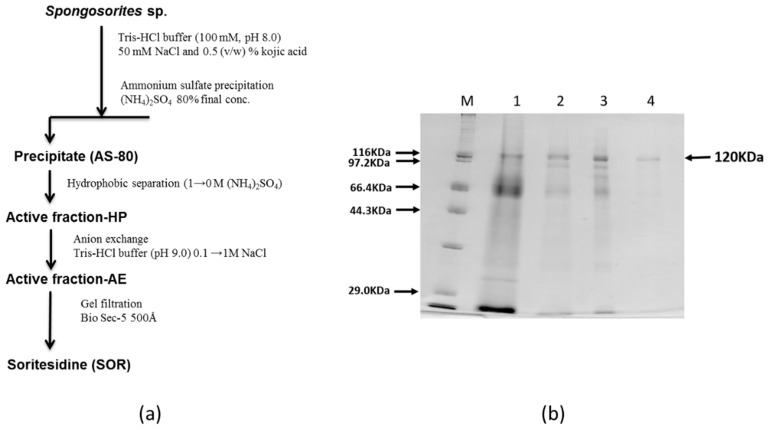
(**a**) Purification scheme of SOR, and (**b**) SDS-PAGE at each step; M, size marker; 1, AS-80; 2, active fraction-HP; 3, active fraction-AE; 4, soritesidine.

Above chromatographic behavior of the active protein in hand, we next largely simplified the separation scheme to enable rapid large scale separation of the extract. Thus the crude extract was first precipitated by ammonium sulfate; in that, the 80% precipitate (fraction denoted as AS-80) was clearly showing a band at 120 kDa (Figure 3). AS-80 was first separated on a Butyl TOYOPEARL 650 M open column followed by a RESOURCE-Q anion exchange column. The active fraction contained the 120 kDa band with some impurities. The final purification was carried out by using BioSec5 (500 Å) HPLC gel filtration column to afford the active fraction with apparently single 120 kDa band (Figure 2S). This result indicated that the 120 kDa band was the active principal of this sponge. We therefore named this protein soritesidine (SOR) after the genus of the sponge. 

### 2.3. Physico-Chemical Properties and Partial Amino Acid Sequences of SOR

**Figure 4:**
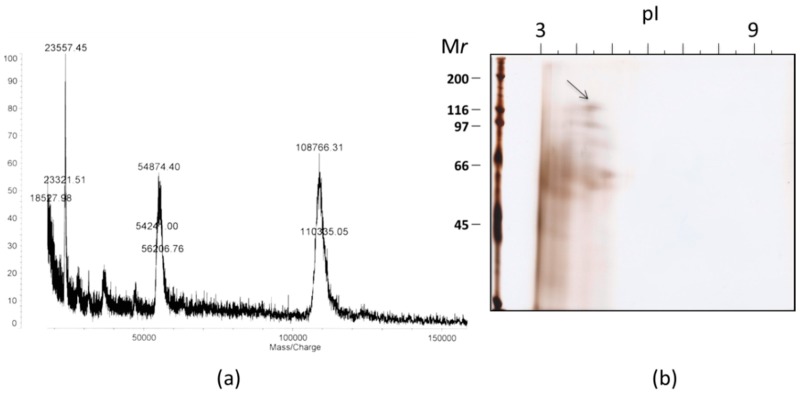
(**a**) A MALDI-TOF mass data for SOR, and (**b**) a two dimensional gel electrophoresis of AS-80. An arrow indicates a spot for SOR.

A MALDI-TOF mass spectra of SOR had a singly charged ion cluster centered at *m*/*z* = 108,766 accompanied by doubly charged ion at *m*/*2z* = 54,874) (Figure 4). Those ions were consistent with the molecular size estimated by SDS-PAGE. In a two-dimensional electrophoresis, a band for SOR migrated around isoelectric point (pI) of approximately 4.5. We next analyzed amino acid sequences of SOR. *N*-terminal amino acid sequence up to 25th amino acids, KLGDQRQIDIASWNTFDFGGVXKAN, was determined by Edman degradation. An in-gel enzyme digest was performed to determine inner sequences of SOR by treating the 120 kDa-band cut out from the SDS-PAGE with lysyl endopeptidase. A total of 26 peptides, including a few large internal peptide fragments, were isolated by HPLC and sequenced (Table S1). Protein BLAST (BLASTP) (BLASTP) search for those sequences against non-redundant protein sequences did not result in significant match with the E-value of less than 0.001. These results indicated that SOR is a previously uncharacterized protein. 

### 2.4. Biological Activities of SOR

**Figure 5:**
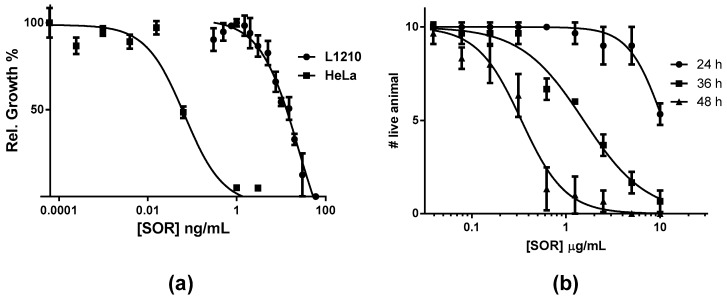
Concentration dependent inhibition by SOR; (**a**) against mouse lymphoma cell line L1210 and human tumor cell line HeLa, (**b**) against brine shrimp larvae at three different observation periods.

**Figure 6:**
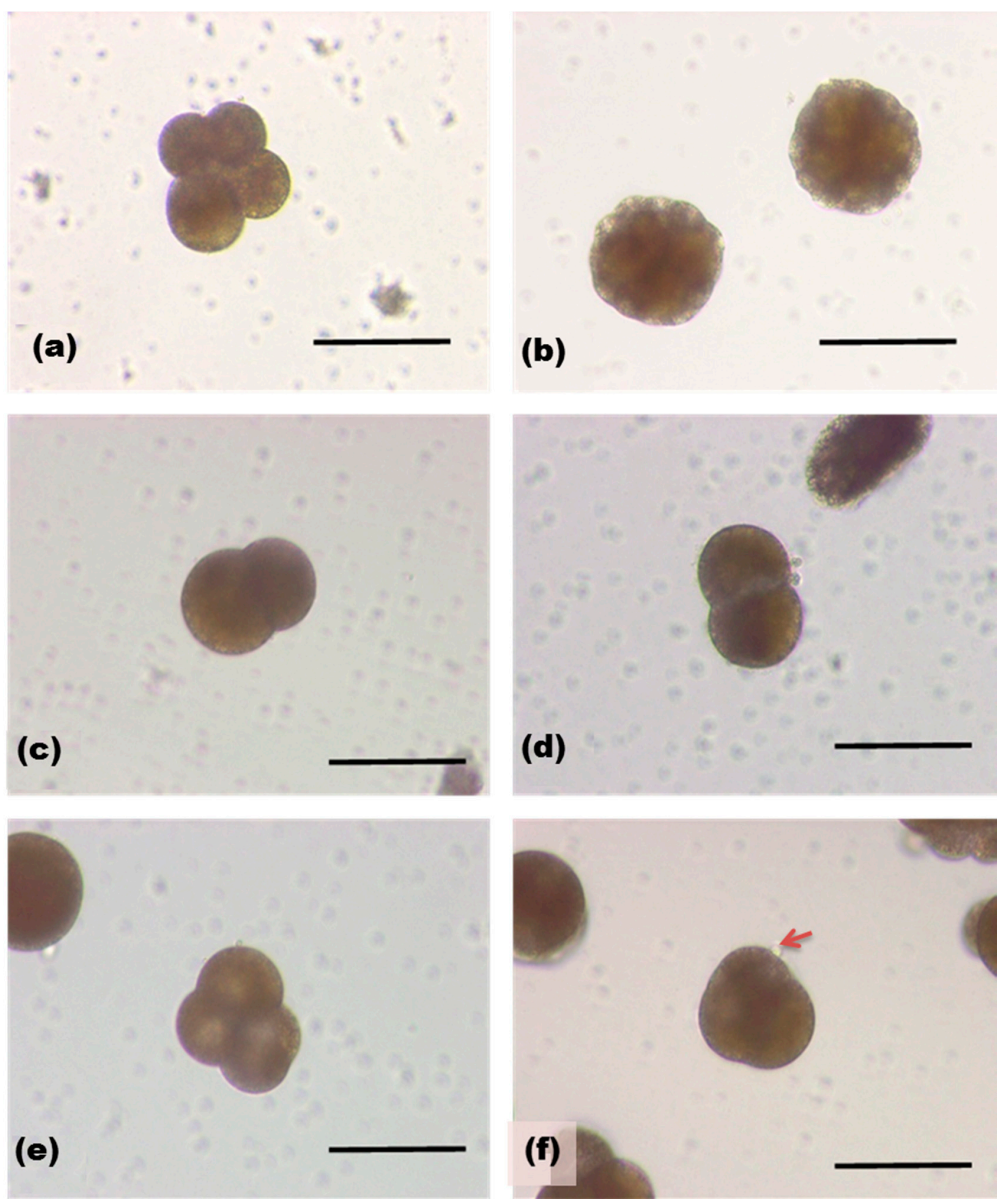
Sea hare eggs 6 h (**a**) and 24 h (**b**) after fertilization. Those treated with SOR (1.3 µg/mL, **c**,**d**), and with 1.3 ng/mL (**e**,**f**). An arrow indicates a bubble-like protrusion on the surface of egg.

**Figure 7:**
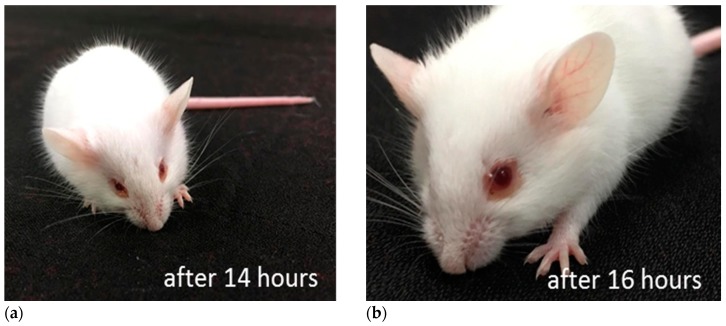
A mouse received SOR via i.c.v. Mouse losing activity 14 h after administration. (**a**) Red ring around eyes started to build up. (**b**) Red ring became conspicuous after 16 h.

Extracts and fractions that contained SOR exhibited cytotoxicity (Table 1), and toxicity over a few different species of animals. To determine overall characteristics of biological activity of SOR, we used cultured tumor cells lines, brine shrimp (Figure 5), sea hare eggs (Figure 6), and mice (Figure 7). In addition, hemagglutination and antimicrobial activities were tested. SOR showed very potent cytotoxicity against HeLa and L1210 murine leukemia cells. IC_50_ values for each cell line, determined to be 0.062 and 12.11 ng/mL (0.517 and 110 pM), respectively, differed significantly being HeLa cells about 200-fold more sensitive than L1210 cells. SOR also showed characteristic toxicity to brine shrimp larvae as was observed in the crude extract and active fractions as follows. The toxicity of SOR was slow onset; that is, toxicity was observed 24–36 h after inoculation of the toxin. The onset time depended on concentration but no earlier than 20 h at all concentrations tested. Concentration-response curves were obtained with the datasets observed at 24, 36, and 48 h after inoculation of nine concentrations of SOR. LC_50_ values were determined to be 10.7, 1.5, and 0.34 µg/mL, respectively. Some cytoskeleton-targeting drugs which are known to be highly toxic to brine shrimp were also tested for comparison (Table 3). The activity of SOR was found to be the most potent among the tested drugs. 

We next tested the effect of SOR on development of fertilized sea hare eggs [23]. Fertilized eggs of *Aplysia kurodai* were removed from egg capsule and developed in sea water. SOR induced concentration-dependent change in the egg morphology (Figure 6). Control egg was in the second cleavage at 6 h after the fertilization, and developed into morula at 24 h. High concentration of SOR (1.3 µg/mL) inhibited the second cleavage as only two blastomeres were observed. Egg development progressed with a low concentration of the protein (1.3 ng/mL), however, overall shape of the cells was shrunken and bubble-like protrusions on the cell surface was often observed.

Toxicity in mice was assessed by injecting the protein i.c.v [24]. Although no acute toxicity was observed, very potent behavioral toxicity developed over time. For example, injection of 574 ng of protein resulted in loss of voluntary movement at 14 h after injection. The mouse lost normal posture at 17 h and died at 18 h. Notably, reddish ring around eyes was observed in those injected the toxin. Lethal toxicity was observed even when 5.74 ng/mouse was administrated if observation was prolonged over 48–54 h after the injection. 

Despite the potent toxicities to animals and animal cells, SOR did not show any hemagglutinating/hemolytic activity against rabbit erythrocyte or an antimicrobial activity against both Gram positive and negative bacteria. 

### 2.5. Possible Mechanism of Toxicity of SOR

**Figure 8:**
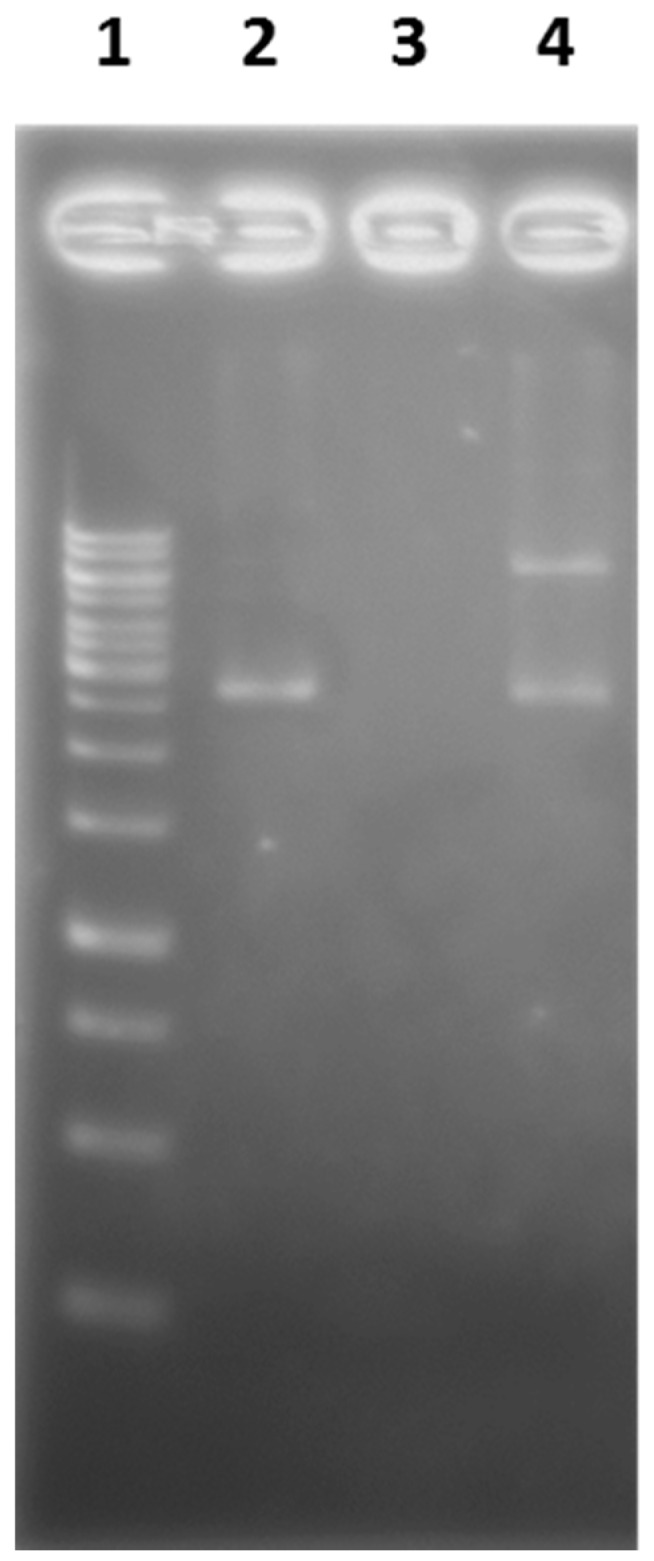
Cleavage of plasmid DNA upon treatment by SOR; line 1, size marker, 2, plasmid DNA, 3, plasmid DNA + SOR, 4, plasmid DNA + SOR + EDTA, a band with lower mobility is a nicked plasmid.

Above biological profiles of SOR suggested possible mechanisms of toxicity. A proteinous toxin SOR would once bind to cell surface through an interaction with unknown receptor molecule, probably through binding with cell surface receptor. The toxin can be translocated inside the cell by endocytosis, escape from the endosome and interact with target molecules in the cell. This route of cellular action is common in bacterial toxins categorized as AB-toxins [25,26]. Most of these toxins are composed of several subunits with discrete functions. The subunit with enzymatic activity (A-subunit) to cause damage in cells is associated with the subunits that translocate the protein into the cells after binding to cell surface receptor (B-subunit). Thus these toxins are collectively called AB_x_-toxins, where x is the number of B-subunits. Examples of those include cholera toxin (AB_5_) [27], Shiga toxin (AB_5_) [28], pertussis toxin (AB_5_) [29], and cytolethal distending toxin (CDT) (AB_2_) [30], diphtheria toxin (single polypeptide, but cleaved by protease to work as AB) [31], as well as plant toxin ricin (AB) [32]. Interestingly, as the distended cell morphology of cells upon treatment by SOR resembled that of CDT, we suspected that SOR shared cellular mechanisms in common with CDT [33]. As the A-subunit of CDT is DNase-I, it can cleave double strand DNA in the nucleus, leading to arrest of the cell cycle at G2/M, causing the cell to distend through a unique signaling cascade to promote actin stress fiber formation [34]. We thus tested the ability of SOR to cleave DNA. When a plasmid DNA was treated with SOR it was cleaved completely, whereas addition of ethylenediaminetetraacetic acid EDTA suppressed the action of SOR, leading to a partially cleaved (nicked) plasmid (Figure 8). These results indicated that SOR has the ability to cleave DNA but the activity depends on a bivalent metal ion. 

## 3. Discussion

In the present study we discovered an extraordinary potent toxin soritesidine (SOR) from the marine sponge *Spongosorites* sp. We developed a relatively simple separation scheme for isolation of SOR after numerous trials. SOR was determined as a single polypeptide with molecular weight about 110 kDa on the basis of its MALDI-TOF mass data. SOR, however, migrated around 120 kDa by SDS-PAGE, and 145 kDa in the gel filtration chromatography. Slightly larger molecular size in the latter cases suggested that the molecular shape of SOR differs in some extent from those of the standard proteins [35]. Nevertheless, these results suggested that SOR present as a monomer in solution. The toxicity of SOR was unusual, characterized by its high potency and slow onset. The potency was so high in terms of cytotoxicity and mice lethality, and the latter may surpass that of palytoxin [36] and maitotoxin [37], the most potent marine toxins known. Of note, the sea wasp toxin (CrTX-A), a well-known potent proteinous toxin, was far less potent than SOR in mice lethality [38] (Table 3). Slow onset of SOR resulted in death of cells or animal 24–48 h after administration. In the brine shrimp assay, we often observed gastric discharge in dead animals. We did not find similar morphological phenotypes after treatment with several known small molecule cytotoxic compounds. We also observed a red ring around the eyes in mice. When we observed sea hare eggs treated by SOR, inhibition of cell division and development was observed. Absence of antimicrobial activity may suggest that SOR cannot permeate the bacterial cell membrane. No hemolytic activity observed for SOR indicated that it is not a plasma membrane disrupting, or channel forming agent. SOR did not have lectin-like hemagglutinating activity. These results overall suggest that the mode of activity of SOR is not categorized to those of antimicrobial peptides, hemolytic proteins, or lectins, although these proteins are often used as innate immunity or chemical defense in various organisms. We found however, that SOR can cleave DNA in a metal ion dependent manner. This result strongly suggested that SOR is a chimeric protein that contains deoxyribonuclease (DNase)-like region in the molecule. Because neither *N*-terminal amino acid sequence nor internal sequences matched to the sequences of known proteins in database, the DNase-like domain might be buried in the inner region of the polypeptide, or differ significantly from the known DNases. DNases are ubiquitous enzymes occurring both in eukaryotes and prokaryotes, functioning in DNA catabolism. DNase I is a metal ion (Ca^2+^, Mg^2+^) dependent endonuclease whereas DNase II can cleave DNA without the presence of metals. Cytolethal distending toxins (CDTs) are equipped with DNase I as one of the three subunits (B-subunit). Although the DNase I itself does not have ability to penetrate cell membrane, two other subunits (A- and C-subunits) play roles in binding onto the cell surface and translocating the enzyme to the target nucleus. In the case of SOR, the present result suggests that it is a single polypeptide with DNase domain. A metal ion dependency of the activity of SOR suggests that the enzyme has DNase I-like property. Taken together, we propose that SOR is the second proteinous toxin with DNA cleaving action, the first being CDT. However, the single polypeptide nature of SOR makes this protein distinct from CDT; thus the SOR should bind to the cell surface and translocate itself to the cell nucleus where DNA cleavage takes place.

## 4. Materials and Methods

### 4.1. General Procedure

Middle pressure chromatography or fast protein liquid chromatography FPLC was performed using ÄKTA FPLC system (GE healthcare bioscience, Uppsala, Sweden). HPLC was conducted on a system comprised of high pressure gradient pumps (PU-980) and photodiode array detector 4015 (JASCO, Tokyo, Japan). Matrix-assisted laser desorption (MALDI) time-of-flight (TOF) mass spectrum was obtained on AXIMA-CFR plus (Shimadzu, Kyoto, Japan) using sinapinic acid as a matrix. Amino acid sequences were analyzed by automated Edman degradation using a gas-phase PPSQ-21A sequencer (Shimadzu). Ultrafree-MC (30,000 NMWL Filter unit, Merck KGaA, Darmstadt, German) was used for ultrafiltration of sample. Cellulose tube (MW 15,000 cut) was used for dialysis. Protein concentrations were determined by BCA protein assay kit (Thermo Fisher Scientific, Waltham, MA, USA) with γ-globulin as a standard. To determine the concentration of purified SOR for biological evaluation, known concentrations of bovine serum albumin (BSA) was stained on SDS-PAGE by Coomassie Brilliant Blue. The bands with different concentration were scanned and the image was quantified by using ImageJ software to obtain a standard curve. The concentration of SOR was estimated by using the curve.

### 4.2. Sponge Specimen

Sponge was collected in Iriomote, Okinawa, Japan at 24.35N, 123.72E, −6 m depth by SCUBA. Each 200 and 740 g of sample was collected in 2002 and 2006, respectively, and the samples were kept frozen pending extraction. The specimen was identified to be *Spongosorites* sp. (Class: Demospomgiae, Order: Halichondrida, Family: Halichondriidae) and registered as QM G324170. The identification was carried out by Dr. John Hooper at Queensland Museum, Australia. 

### 4.3. Bioassays

#### 4.3.1. Brine Shrimp Assay

Commercially available dried eggs of brine shrimp (*Artemia salina*) were incubated in artificial sea water (ASW) at 25 °C for 24 h. A total of 10 hatched larvae were placed in a well of 24-well plate with brine (2 mL). The diluted test sample (20 µL) was added to the well and the plate was kept at 25 °C. The mobility of the animals was observed at every 12 h period for 48 h to count number of live animals. An individual without any motion during the observation was regarded as dead. Three wells were used for one sample and the average number of dead animals at each concentration was plotted to generate a concentration–response curve which was analyzed by using Prism software (GraphPad, San Diego CA) to obtain LC_50_ values. 

#### 4.3.2. Sea Hare Egg Assay

Sea hare egg assay was carried out by using the eggs collected from newly deposited egg mass of sea hare (*Aplysia kurodai*). The egg was removed from the egg mass and cut in pieces in ASW. The eggs were collected and gently centrifuged by hand centrifuge apparatus. The precipitated eggs were rinsed by ASW twice and transferred to 24-well plate. Filtered natural sea water was added to each well (2 mL). The test sample (10 µL) and then the egg suspension (20 µL) were added. Egg development was observed under microscope every few hours. 

#### 4.3.3. Mouse Behavioral Assay

The mouse behavioral assay was performed under approval by the Ethical Committee of Experimental Animal Care at Hokkaido University. The effects of samples on mice behaviors were assessed as described previously. Briefly, an aqueous solution of sample (10 µL) was injected i.c.v. in male ddY mice of 3 to 4 weeks old (Japan SLC Inc, Hamamatsu). Changes in behaviors and appearance of the animal were observed for one week. 

#### 4.3.4. Cytotoxicity Assay

Cytotoxicities of the crude extract against HT29 (human colorectal adenocarcinoma), A549 (human lung carcinoma), and MDA-MB 231 (human breast carcinoma) cells were determined for initial screening by PharmaMar SA. Cells (5 × 10^3^ cells/well) were inoculated into a well of 96-well microtiter plate and pre-incubated for 18 h. Sample dissolved in DMSO:H_2_O (3:7) was added (5 µL) to the well and incubated for 72 h. The cell was fixed by adding trichloroacetic acid (50%) and then stained with 0.4% sulforhodamine. OD_490_ was measured to quantify survived cells. The activity (A) was indicated using the following equation:

A = 100 × (T − T_0_)/(C − T)

where T_0_ is the OD before the incubation, T is OD at the end of incubation, and C is OD of the control well. Thus cytostatic activity was shown as positive value and cytotoxicity as negative value. L1210 (RBRC-RCB2844, Riken), and HeLa (RBRC-RCB0007) cells were used. Culture media used for each L1210 and HeLa cells was RPM1640 and EMEM, respectively, with 10% fatal bovine serum (FBS) for all the media (Wako Pure chemicals, Osaka). Cells were pre-cultured using a CO_2_ incubator (37 °C, 5% CO_2_) for 3 days (L1210) or 7 days (HeLa). For L1210, confluent cells (1 × 10^5^ cells/mL) were inoculated (50 µL) to each well of 96-well microtiter plate. The sample dissolved in the medium (200 µg/mL) was filtered (0.22 µm) and serially 2-fold diluted, and then added (50 µL) to each well so that the final concentration of sample between 50 and 0.024 µg/mL, and cell 5000 cells/well, were archived. For HeLa, confluent cells (2 × 10^4^ cells/mL) were inoculated (100 µL) to the well and pre-incubated for 24 h. Sample (10 µL) was added to the well (final concentration of sample 50–0.024 µg/mL) and incubated for 48 h. Growth of cells was quantified using a cell counting kit-8 (WST-8, Dojindo Laboratories, Kumamoto, Japan) following manufacturer’s protocol. Absorption at 450 nm was measured using microplate reader. Mean triplicate of the data was plotted to generate a concentration–response curve and was analyzed using Prism software.

### 4.4. Extraction and Separation

#### 4.4.1. Extraction and Initial Separation

The sponge specimen (IRI-155, 10 g frozen) was extracted with water (10 mL × 3 times) at room temperature and the lyophilized material was chromatographed on Sephadex LH-20 open column (2.5 × 120 cm, GE healthcare) using water as eluent (flow rate 0.5 mL/min). Fractions (10 mL/fraction) were combined according to TLC (butanol-acetic acid-water 4:1:1) into a total of 38 fractions. Activity of each fraction was monitored by using brine shrimp assay. Initial evaluation of cytotoxicity of the extract and the fractions were carried out by PharmaMar SA using three cell lines HT-29, A-549, and MDA-MB-231.

#### 4.4.2. pH Dependency Assay

The sponge specimen (IRI-155, 890 mg frozen) was extracted with Tris-HCl buffer (100 mM, pH 8.0) containing 200 mM NaCl and 0.5% (*w*/*v*) kojic acid. The extract was passed through HiTrap Desalting (5 mL, GE healthcare) eluting with 0.6 M NaCl to give a solution of crude extract. This crude extract (250 µL) was mixed with 50 µL of buffer (0.5 M sodium citrate, pH 4.0, 5.0, 6.0; 0.5 M HEPES, pH 7.0; 0.5M Tris-HCl, pH 8.0, 9.0; 0.5M glycine, pH 10.0) and distilled water (200 µL), and plated on 24-well assay plate. The plate was incubated in an ice-water bath for 2 days, and then used for brine shrimp assay. Observation was made at 36 h after inoculation of the animals. 

#### 4.4.3. Small Scale Extraction

Separation of SOR sponge specimen (IRI-155, 100 g) was extracted with 300 mL of Tris-HCl buffer (100 mM, pH 8.0) containing 200 mM NaCl and 0.5 (*w*/*v*) % kojic acid. To the crude extract (37. 5 mL), 4 M ammonium sulfate (12.5 mL) was added. This solution (50 mL) was charged on HiTrap Butyl FF column (5 mL, GE healthcare). The column was washed by passing 1 M ammonium sulfate through the column until the absorbance at 280 nm (Abs280) becomes less than 0.05 AU. Then the column was eluted by a gradient of 1 M ammonium sulfate and water. The active fraction was combined and desalted using HiTrap desalting 26/10 (1 mL, GE healthcare) eluting with Tris-HCl buffer (100 mM, pH 8.0). The protein fraction (58 mL) was charged to RESOURCE-ISO (1 mL, GE healthcare) column. The column was eluted by a gradient of 2 M ammonium sulfate and water. The active eluent from the above column was desalted and then proteinous eluate (42 mL) was charged directly to a RESOURCE-Q (1 mL, GE healthcare) anion exchange column. The absorbed proteins were separated by eluting with a gradient of Tris-HCl buffer (100 mM, pH 8.0, 0.1-0.4 M NaCl). For size exclusion chromatography, the active fraction from the ion exchange separation was concentrated by ultrafiltration to 100 µL, and then it was separated on a Superdex 200 10/300 GL column (GE healthcare) eluting with 50 mM Tris-HCl buffer (pH 9.0, containing 0.2 M NaCl). The standard protein mixture consisting of glutamate dehydrogenase (290 kDa), lactose dehydrogenase (142 kDa), enolase (67 kDa), myokinase (32 kDa), and cytochrome C (12.4 kDa) was used. 

#### 4.4.4. Large Scale Separation

For large scale batch separation, sponge specimen (IRI-155, 2006 collection, 620 g) was extracted as above to give a total of 7 L of the crude extract. Ammonium sulfate was added to the extract at 0 °C to a final concentration of 80%. The mixture was stirred at 0 °C for 1 h and then centrifuged (50 min at 8500 rpm, 4 °C). The precipitation was re-dissolved in 50 mM Tris-HCl buffer (pH 8.0) containing 1 M ammonium sulfate. This material (SOR1-56-2) was denoted as AS-80 which was used for further large scale separation. AS-80 was first separated on an open Butyl Toyopearl 650M gel (TOSOH Tokyo, Japan) column. The gel (100 mL) was suspended in 50 mM Tris-HCl (pH 8.0) with 1 M sodium sulfate. The extract solution (100 mL) was absorbed by starring with the gel for overnight. The gel preparation was packed in a column. The absorbed proteins were eluted (1 mg/mL) stepwise with 0.9, 0.7, and 0.2 M sodium sulfate-containing buffer until Abs280 become less than 0.05 AU for each eluent. The active fraction eluted with 0.2 M sodium sulfate-containing buffer was dialyzed against 50 mM Tris-HCl (pH 9.0 with 0.1M NaCl) and was absorbed on a RESOURCE-Q (1 mL) column. The absorbed proteins were eluted with a gradient of Tris-HCl buffer (100 mM, pH 8.0, 0.1–0.6 M NaCl). Final purification of SOR was performed on an HPLC using BioSec 5 (500 Å, Agilent Technology, Santa Clara, CA, USA) eluting with 50 mM Tris-HCl, pH 8, containing 0.2 M NaCl. 

#### 4.4.5. Sodium Dodecyl Sulfate-Polyacrylamide Gel Electrophoresis (SDS-PAGE)

SDS-PAGE was performed in a 5, 8, 15%, or 8–15% gradient polyacrylamide gel. The gel was stained with zinc reverse staining by immersing gel in 0.2 M imidazole/0.1% SDS for 10 min, then with 0.2 M zinc sulfate. The staining reaction was quenched by rinsing with water. Alternatively, 0.25% Coomassie Brilliant Blue was used to stain protein bands. 

Two-dimensional polyacrylamide gel electrophoresis (2-DE) was carried out according to the manufacturer’s protocol. 443 µg of sample was mixed with a rehydration solution (8 M urea, 0.5% 3-[(3-cholamidopropyl)dimethylanmonio]-propanesulphonic acid (CHAPS), 0.5% Bio-Lyte 3/10 (Bio-Rad, Hercules, CA, USA), 0.2% dithiothreitol, 0.002% bromophenol blue). The solution was applied to IPG strip 3–6 (BioRad). Isoelectric focusing was performed at 20 °C as follows: 12 h rehydration, 30 min at 300 V, 30 min at 1000 V, 2 h at 5000 V. After the focusing, the strip was equilibrated in 2% SDS, 50 mM Tris-HCl, pH 8.8, 6 M urea, 30% glycerol, 0.25% DTT for 15 min with shaking. Then it was re-equilibrated with 2% SDS, 50 mM Tris-HCl, pH 8.8, 6 M urea, 30% glycerol, 4.5% iodoacetamide for 15 min with shaking. The strip was placed on the 12% polyacrylamide gel, and second electrophoresis was performed same as SDS-PAGE. The gel was stained by using silver stain kit (Wako Pure Chemical).

#### 4.4.6. Amino Acid Sequence Determination

Edman degradation for the *N*-terminal and enzyme digests of SOR was carried out using automated gas phase protein sequencer PPSQ-21 (Shimadzu). A sample of semi-pure SOR was separated on a SDS-PAGE, blotted to PVDF membrane, and then the membrane was stained with Ponceau S solution. The membrane with a band at 120 kDa was cut and subjected to *N*-terminal sequence analysis. For internal amino acid sequence analysis, the 120 kDa band on the membrane was reduced by dithiothreitol and then treated with iodoacetic acid and 1 M NaOH (10 µL) for 20 min. The membrane was thoroughly washed with water then with acetonitrile (2%), and finally immersed in a buffer (20 mM Tris HCl with acetonitrile 70%). Finally *Actomobacter* protease I (Wako pure chemical, Tokyo, 1 µg in 50 µL buffer) was added and the mixture was stood overnight at room temperature. The digest was separated on a reversed-phase HPLC (Cadenza CD-C18, Imtakt, 0.05% TFA-acetonitrile 0.05%, gradient). Each peak (215 nm) was concentrated and dissolved in 5 µL of water and the same amount of MeOH. The enzyme digest was absorbed on a PVDF membrane for the sequence analysis.

#### 4.4.7. Cleavage of Plasmid DNA by SOR

SOR containing fraction (SOR 3-62-5) from the hydrophobic separation was used for the assay. A plasmid DNA (4 ng) was mixed with SOR 3-62-5 (0.0088 mg/mL), or an addition of EDTA (12.5 mM) in Tris-HCl buffer (pH 8.3) containing 0.1 M NaCl. The mixture was stood for 24 h at room temperature and separated by agarose electrophoresis using GeneRuler 1 kb DNA Ladder (Thermo scientific) as a marker, stained with ethidium bromide (15 min), and then observed under UV (260 nm) light. 

## 5. Conclusions

In conclusion, the present study revealed that SOR is an extraordinary potent proteinous toxin which may have an AB-toxin-like structural motif. The cellular response to SOR as well as DNA cleaving action by SOR suggest that it is a novel type of genotoxin. Obviously gene/amino acid sequences, structure, and mode of activity for SOR are of great interest and studies regarding these issues are in progress in our laboratory.

## Figures and Tables

**Table 1:** Cytotoxicity of extract and fraction of *Spongosorites* sp.

	HT-29	A-549	MDA-MB-231
Crude extract	20 *^a^* (5)	−26 *^b^* (5)	−1 *^b^* (5)
Fraction 6 *^c^*	27 *^a^* (25)	−1 *^b^* (5)	−3 *^b^* (5)

*^a^* Cytostatic action, % growth of control. *^b^* Cytotoxicity, % inhibition. *^c^* One of the active fractions of Sephadex LH-20 separation. Concentration (g/mL) is shown in parenthesis.

**Table 2:** Purification Table of the crude extract of *Spongosorites* sp.

Fraction	Total Protein (mg)	Total Activity (unit) *^a^*	Recovery (%) *^b^*	Relative Activity Unit/mg
Crude extract	2400	556,000	100	230
HiTrap	294	234,000	24	796
RESOURCE-ISO	32	15,400	2.8	481
RESOURCE-Q	2.5	18,500	3.3	7370
Fraction A	0.14	19,400	3.5	137,588

*^a^* A unit represents the concentration that kills 50% of brine shrimps in 36 h in the brine shrimp assay (see Materials and Methods). *^b^* (unit of interest/unit of crude extract) × 100.

**Table 3:** Toxicities of SOR, some cytotoxic compounds, and marine toxins.

Compound	MW	Brine Shrimp LD_50_ *^a^* (µg/mL, µM)	Mice Lethality ng, pmol/ kg	Cytotoxicity
SOR	116,000	0.34, 3.1 × 10^−3^	287, 2.39 (i.c.v.)	0.517 pM *^b^*
Paclitaxel	854	0.86, 0.99		2.6 nM *^b^* [18]
Cytochalasin D	507	0.29, 0.34		0.1 µM *^c^* [19]
Swinholide A	1389	0.55, 0.40		22 nM *^d^* [20]
Palytoxin	2680		45, 16.8 (i.v.)	~0.7 pM *^e^* [21]
Maitotoxin	3425		130, 38 (i.p.)	~58 pM *^f^* [22]
CrTX-A	43,000		20,000, 465 (i.v.)	

*^a^* Recorded at 48 h observation period. ^*b*^ HeLa cells, *^c^* HeLa cells, measured as zeiosis formation. *^d^* L1210 cells, *^e^* A549, *^f^* rat aortic smooth muscle cells.

## Supplementary Materials

The following are available online at https://www.mdpi.com/1660-3397/17/4/216/s1, Figure S1: Chromatograms for the separation of SOR. Figure S2: HPLC separation of SOR. Table S1: Sequences for inner peptides obtained from SOR.
