# Supplementary material for: Soritesidine, a Novel Proteinous Toxin from the Okinawan Marine Sponge Spongosorites sp
Source: Mar Drugs. 2019 Apr 8;17(4):216. doi: 10.3390/md17040216 (PMC6520796; doi:10.3390/md17040216)
Supplement: Supplementary file 1 [file marinedrugs-17-00216-s001.pdf]

Supplementary Materials

Soritesidine, a novel proteinous toxin from the Okinawan marine sponge *Spongosorites* sp.

Ryuichi Sakai[1*], Kota Tanano[2], Takumi Ono[2], Masaya Kitano[1], Yusuke Iida[1], Koji Nakano[1], and Mitsuru Jimbo[2]

[1] Hokkaido University, Faculty and Graduate School of Fisheries Sciences; ryu.sakai@fish.hokudai.ac.jp
[2] Kitasato University, School of Marine Bioscience; mjinbo@kitasato-u.ac.jp
* Correspondence: ryu.sakai@fish.hokudai.ac.jp; Tel.: +81-138-40-5552

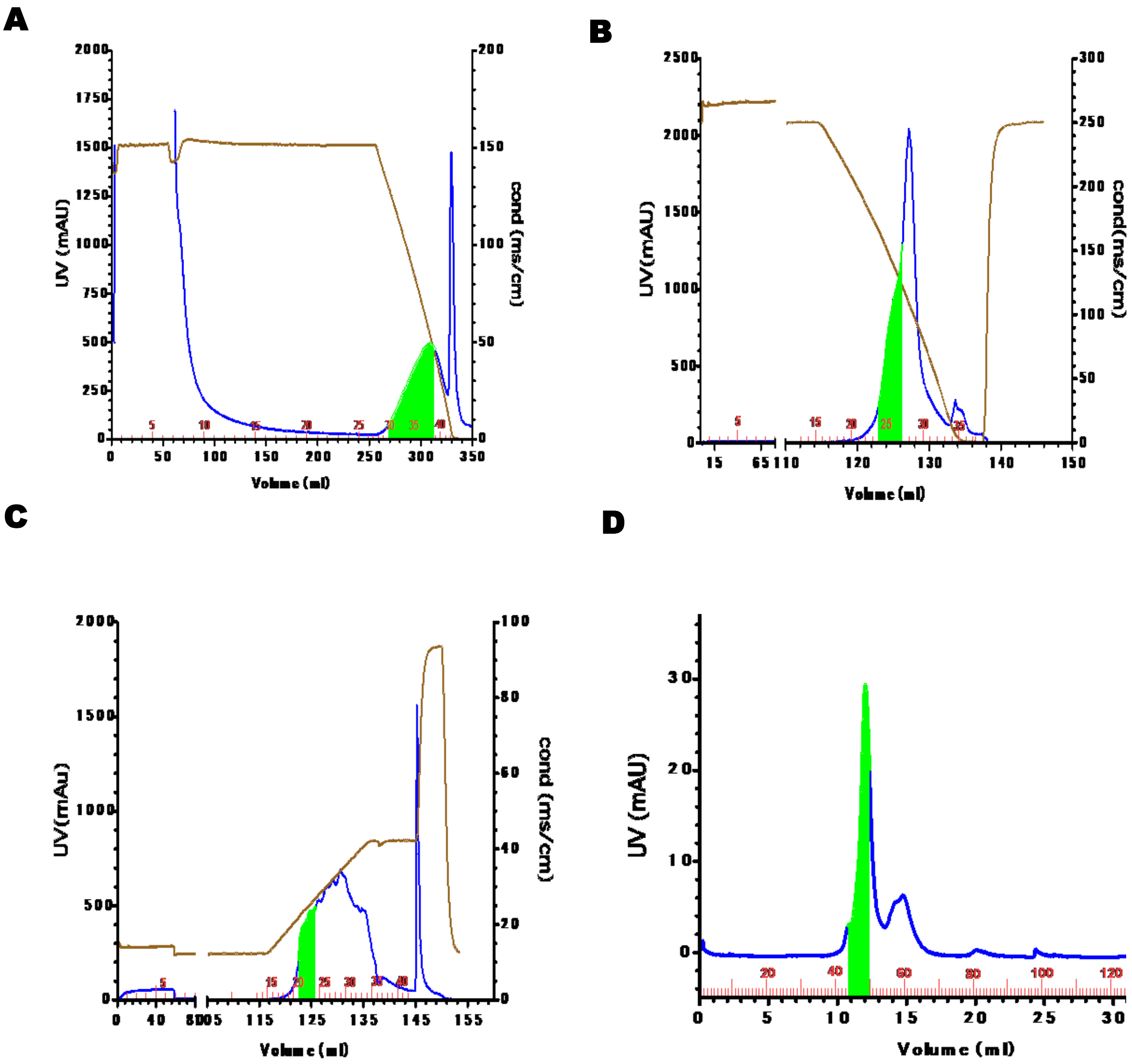

Figure S1. Chromatographic behaviors of SOR in the sponge extract. The first hydrophobic chromatography using HiTrap Butyl-FF (A), the second hydrophobic chromatography by RESOURCE-ISO (B), An anion exchange chromatography by RESOURCE-Q (C), and Gel filtration chromatography on a Superdex 200 10/30 column.

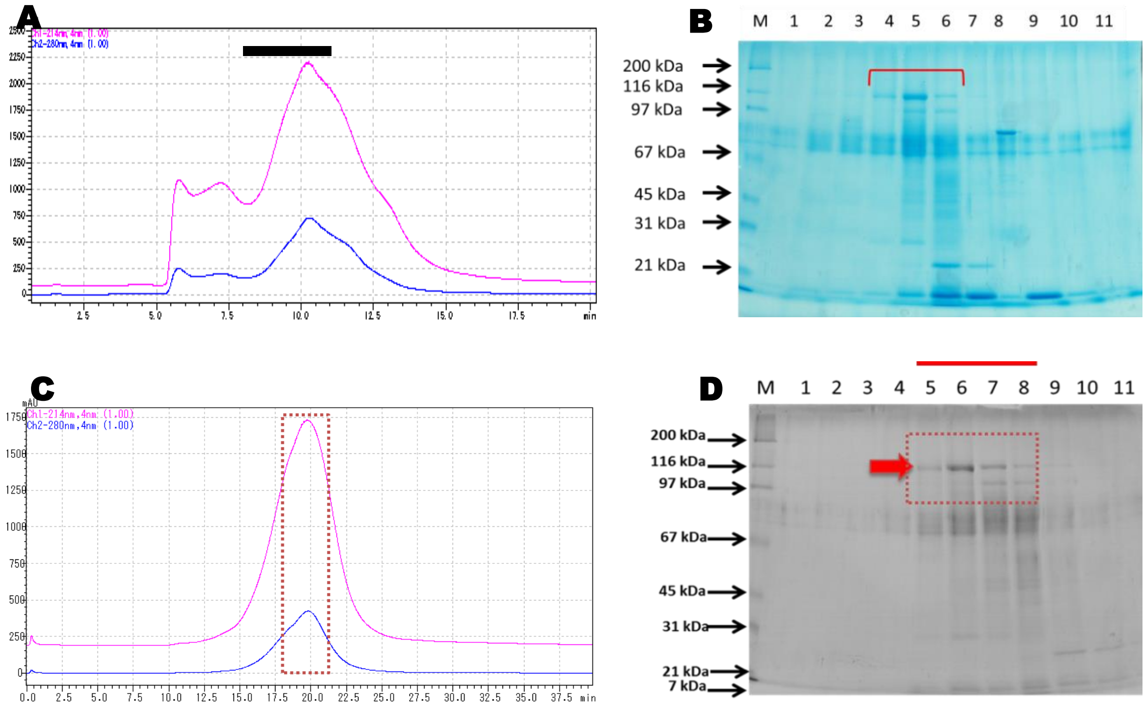

Figure S2. Purification of SOR by BioSec-5 gel filtration HPLC. The first run (A) and SDS-PAGE of fractions (B). The second run (C) and SDS-PAGE of fractions (D). Bar indicates fractions with brine shrimp activity.

Table S1. Amino acid sequence for N-terminal and protease digests of SOR determined by Edman degradation.

Partial amino acid sequences
KLGDQRQIDIASWNTFDFGGVXKAN (N-terminal)
(K)XGH
(K)LPG
(K)NFDY
(K)SGXST
(K)TGAXXXE
(K)ESAAETEN （E/G）
(K)(A/G)GXNNNHXH
(K)PLDVRGTY（D/E） XXXV
(K)ASAAPTNN（N/A） XTSLSSGXD（E/G）
(K)HXQDRIQPAXPPXH
(K)XLD（D/E） ETLE
(K)ANTGIGNVVIERTDNPNTVPYIPA
(K)LALEVPLRTVNXT
(K)FAVITLGDLNADGXH
(K)GDGENXNDNXDXYD
(K)ASTGSTIPXGXXT
(K)（Q/G） AGFVPNXTXDXT
